# Impact of ivermectin on nerve regeneration following sciatic injury in mice: the consequences of dietary high fructose

**DOI:** 10.55730/1300-0144.5971

**Published:** 2024-12-19

**Authors:** Ezgi Deniz ARIKAN, Barışcan ÇİMEN, Ayşe Ece GEZEN YILMAZ, Elif AKAYDIN, Berkay ALPAY, Deniz Ekin ERBAŞ, Erblina NIKSHIQI, Sevda F. MÜFTÜOĞLU, Yıldırım SARA

**Affiliations:** 1 Department of Medical Pharmacology, Faculty of Medicine, Hacettepe University, Ankara, Turkiye; 2 Department of Histology and Embryology, Faculty of Medicine, Hacettepe University, Ankara, Turkiye

**Keywords:** Sciatic nerve, nerve regeneration, high fructose corn syrup, ivermectin

## Abstract

**Background/aim:**

Peripheral nerve injuries (PNIs) are debilitating disorders affecting predominantly the younger generation, often leading to significant disabilities. Current treatment strategies are inadequate for addressing the complex nature of these injuries. Peripheral nerve healing and functional recovery are crucial components of both pathophysiology and therapeutic approaches. High fructose corn syrup (HFCS) is a sweetener frequently used in several beverages and foods. It is associated with several metabolic disturbances including insulin resistance and may impair nerve healing. This study investigated the therapeutic role of ivermectin on nerve regeneration following sciatic nerve injury and evaluated motor and sensorial functions together with histopathological evaluation. Additionally, we aimed to compare nerve healing between animals that consume HFCS and those that do not.

**Materials and methods:**

Forty-eight male Swiss albino mice were randomly divided into six groups, with three consuming HFCS-42 (11% v/v) and the other three regular tap water for 8 weeks. On day 28, sciatic nerve injury (SNI) was caused in all groups. Ivermectin (1 mg/kg) or gabapentin (30 mg/kg) treatments were administered to selected groups. Body weight, blood glucose, motor function (rotarod, open field test), and thermal–mechanical sensorial functions were assessed weekly. Finally, insulin levels were measured and histopathological samples were taken.

**Results:**

Eight weeks of HFCS consumption impaired mechanical and thermal sensory functions and resulted in histopathologically poor nerve repair. Ivermectin resulted in improved sensorial and faster motor function recovery in the HFCS groups. Elevated plasma insulin levels/HOMA-IR values were diminished by ivermectin in the HFCS groups. In the ivermectin non-HFCS group, histopathology revealed accelerated healing and higher scores in total. Ivermectin also ameliorated mechanical sensation loss after SNI along with cold sensation.

**Conclusion:**

Ivermectin accelerated sensorial and motor nerve recovery, resulting in faster nerve healing alongside improved insulin resistance, suggesting it might serve as a potential foundation for developing a new treatment for nerve regeneration after injury.

## 1. Introduction

Peripheral nerve injuries (PNIs) are an underestimated health problem causing significant disability and reducing quality of life [1]. Trauma is one of the most common causes of PNIs in the general population, primarily affecting younger individuals [2]. The working capacity of adults, who are the most active part of society economically and socially, is affected to a great extent. Frequently encountered signs and symptoms in clinical practice are numbness, paresthesia, and muscle weakness and wasting. Although the incidence of PNIs was given between 1.46% and 2.8% in earlier studies, the latest research reports higher numbers of patients with PNIs likely due to underdiagnosis and insufficient record keeping [2,3]. Sudden stretching of a limb, transection, thermal and electrical insults, radiation, and compression are the primary causative factors [4,5]. Damage to peripheral nerves results in motor and sensory loss of functions alongside deformities and neuropathic pain [6]. The current approach to managing traumatic peripheral nerve lesions involves nerve grafts, surgical repair, conduits, electrical stimulation, and treatment with vascular endothelial growth factor [7]. Nonetheless, the current treatments available are inadequate and primarily invasive and require a limited window of time to be effective [8,9]. Within this critical window, the neuroregenerative process involves essential steps such as Wallerian degeneration, axonal regeneration, and axonal guidance for growth cones and the viability of end organs [10].

The key factor in complete functional recovery and reinnervation is determined by how successful the nerve healing is. In cases in which over 90% of the axons are damaged, axonal regeneration becomes the main route for recovery [10,11]. Creating a microenvironment that supports the regenerative response has been a therapeutic goal for many years. Within this frame, several neurotrophic factors (e.g., nerve growth-factor (NGF), brain-derived neurotrophic factor (BDNF), glial-derived neurotrophic factor (GDNF)), and Schwann cells (SCs) have been utilized to enhance regeneration. Shortly after injury, SCs proliferate and differentiate into nonmyelinating, growth-supporting phenotypes [12]. Subsequent to Wallerian degeneration, dedifferentiated SCs take over, secrete neurotrophs, and produce bands of Büngner by proliferating on the remaining endoneurial tube [10]. Newly formed tubes provide a path for regenerating axons to grow. Insufficiently supported axon branches due to lack of neurotrophs undergo pruning and ultimately degenerate [6]. Axonal regeneration is also impeded by increased oxidative stress, ischemia, inflammation, hyperglycemia, and lack of vascular support [13–16]. Widespread adaptation of a Western diet containing high concentrations of sugars such as high-fructose corn syrup (HFCS) has led to a rise in incidences of cardiovascular diseases and metabolic disorders in the last 50 years. Among them, hyperglycemia, insulin resistance, and type-2 diabetes mellitus are leading contributing factors.

In various diabetic animals with PNI, delay in Wallerian degeneration is observed with an impairment in axonal regeneration [16]. Hyperglycemia causes schwannopathy, implying chronic SC dysfunction, accompanied by decreased release of neurotrophic factors [17]. In addition, an inflammatory environment is produced by hyperglycemia through oxidative stress and increased TNF-α, IL-6, IL-1β, NF-κB, and toll-like receptor (TLR) expression, leading to axonal degeneration and axon-SC contact disorder [18].

Sciatic nerve crush/injury is one of the most used models of peripheral nerve injury in rodents [19]. Crushing the sciatic nerve (axonotmesis) disrupts the axons, but leaves the SC basal laminae intact; by this way the process of nerve regeneration stays optimal and results are reproducible [20,21]. For this reason, this model is widely adopted to investigate peripheral nerve regeneration [22].

Ivermectin (IVM) is a low-cost antiparasitic drug that has a well-established side effect profile, is easy to access, and is approved by major drug evaluation agencies worldwide for the treatment of parasitic infection [23]. Previous studies have shown that IVM acts on glycine and GABA channels [24]. It binds to multiple binding sites on the α1β2γ2L GABA_A_ receptor subtype, exhibiting varying potency and inducing both reversible and irreversible effects on GABA-mediated currents [25]. Furthermore, IVM has been shown to induce the release of GDNF from dermal fibroblasts during wound healing and block the production of proinflammatory cytokines via the NF-KB pathway and TLR-4 expression [26,27]. We selected gabapentin as a positive control for IVN due to its common use in clinics for treating various neuropathic pain conditions resulting from metabolic and traumatic etiologies [28].

Traumatic nerve injuries are debilitating and challenging to treat despite various treatment options. Functional recovery is achieved only via optimal nerve regeneration, which is a quite complex and lesser-known cascade of events. IVM has diverse therapeutically promising effects such as antiinflammatory, neurotrophic, and GABAergic properties. Herein, we evaluated the healing effects of IVM in a crush injury model. HFCS has become indispensable in our lives, yet its effects on peripheral nerve regeneration have not been fully investigated. Using this model, we also examined the effects of HFCS on healing alone and with IVM treatment.

## 2. Material and methods

### 2.1. Animals

Forty-eight male Swiss Albino mice (Kobay AŞ, Türkiye) weighing approximately 22–27 g were used. Female mice were not utilized due to possible confounding effects of the estrous cycle. The mice were housed at a room temperature of 21 °C with a 12-h night and 12-h day cycle. All mice had ad libitum access to food and water during the experiment (except HFCS mice). They were randomly divided into 6 groups. This research was carried out following the standards of ARRIVE and was approved by the Hacettepe University Animal Experimentations Ethics Board (approval no: 2021/0-23).

### 2.2. Experimental drugs

HFCS was given to rodents for investigating insulin resistance, T2DM, metabolic syndrome, steatohepatitis, or intestinal cancers by modifying its formula and treatment periods [29,30]. In the food industry, HFCS contains mostly 42% (HFCS-42) or 55% (HFCS-55) fructose; thus in human and animal studies these two forms are used. In the present study, we adopted a similar methodology from our previous work utilizing the same formula and treatment period [31].

From day 0, the mice were fed drinking water or drinking water containing 11% high fructose corn syrup-42 (HFCS-42 Çemsan, Türkiye). HFCS-42 contains 45% fructose and 54% glucose and has a Brix value (20 °C) of 71.

Gabapentin (30 mg/kg, i.p. Abdi İbrahim, Türkiye) and IVN (1 mg/kg, i.p. Boehringer Ingelheim, Germany) treatments were given daily after the SNI operation.

### 2.3. Experimental protocol

The animals were kept for 10 days for acclimatization and then the experiments were performed. For 8 weeks, 3 groups of mice received tap water and the rest received 11% HFCS solution (Table). After 4 weeks, the right sciatic nerves of all mice were surgically injured. The tap water receiving group of animals was treated with i.p. vehicle, IVN, and gabapentin, consecutively. This drug regimen was similarly applied to the groups receiving HFCS. Body weights and rotarod test and mechanical and thermal sensorial test results were evaluated weekly. At the end of 8 weeks, blood samples were collected and the mice were sacrificed to collect histologic specimens.

### 2.4. Sciatic nerve injury (SNI)

After intraperitoneal injections of ketamine (90 mg/kg) and xylazine (10 mg/kg) anesthesia, the posterior surface of the right leg of the mice was shaved. The skin was disinfected with 10% polyvinylpyrrolidone-iodine solution. A 1-cm-long skin incision was made on the posterior surface of the right leg to expose the sciatic nerve. The right sciatic nerve was compressed by applying constant pressure with hemostatic forceps for 30 s 0.5–1 cm proximal to the point where it branches to the tibial and peroneal nerves, and SNI (crush injury) was created. The left sciatic nerve was opened in the same way, but only surgery was performed without any damage. After the procedure was completed, the skin was closed with 4.0 sutures.

### 2.5. Static sciatic index (SSI)

This test was used to evaluate the functional outcomes of damage and recovery in the sciatic nerves of mice [32]. The mice were placed in a 20 × 12 × 9 cm cage with a transparent bottom and illuminated with a cold light source from below. A camera was placed under the cage and paw width/length and distance between the fingers were measured weekly. In each evaluation, 50 photographs were taken in approximately 10 min per animal. Five of these photographs were selected and used to calculate the SSI. The criteria used to select the photographs were as follows: 1) the mouse was standing straight, 2) all 4 paws of the mouse were on a transparent background. The average of 5 measurements was taken for each mouse. The SSI was calculated using the following formula [32]: SSI = 101.3 × toe spread factor (TSF) − 54.03 × print length factor (PLF) − 9.5. Toe spread (TS): distance between tips of the 1st and 5th toe. Print length (PL): distance between tip of the 3rd toe and the most posterior aspect of the paw in contact with the surface. TS and PL parameters’ factors were calculated with this formula: (injury side parameter – sham side parameter)/sham side parameter. For example, print factor (PLF) = (PL injury side – PL sham side)/PL sham side.

### 2.6. Behavioral experiments

#### 2.6.1. Open field arena (OFA) test

This test was used to evaluate locomotion in mice [33]. The mice were placed in a square chamber (45 × 45 × 45 cm) with glass walls and the distance travelled was recorded for 10 min with a video tracking system.

#### 2.6.2. Rotarod test (RT)

This test was used to evaluate the motor functions of mice [34]. It was determined how long it took the mice to fall from a rod rotating at constant (20 rpm) speed. The experiment was repeated 5 times for each mouse and the fall times were averaged. Before the first test was performed, familiarization of the mice to the apparatus (YSED, Türkiye) was assured.

### 2.7. Plasma insulin level and HOMA-IR

Blood samples were collected to determine the insulin resistance status of the mice. On the last day of the experiment, approximately 1 mL of blood was collected from the femoral vein after urethane anesthesia for histological tissue sampling and sacrifice. It was placed in heparinized tubes for plasma samples. After centrifugation (2000 × *g* for 10 min), the supernatants were stored at −80 °C. Insulin levels were measured using ELISA. The procedure followed the instructions provided by the commercial kit (Bertin Pharma SPIBio, UK, Kit code: A05105).

HOMA-IR was calculated by using 28-day fasting blood glucose (mg/dL) and insulin levels after 12 h of hunger with the formula: HOMA-IR = [fasting insulin (IU/mL) × fasting glucose (mg/dL)]/405 [35].

### 2.8. Histopathologic evaluation

The sciatic nerves of the mice were harvested after sacrifice. The length of the dissected nerve was around 1 cm and that included the anatomical region of the injury at equal distances from both sides. Sciatic nerves were stored in formaldehyde solution at +4 °C for postfixation; tissue tracing was performed afterwards and they were embedded in paraffin. Paraffin blocks were sectioned at 5 μm and deparaffinized and stained with hematoxylin and eosin, Masson’s trichrome, and Luxol fast blue. Immunofluorescence labeling with anti-pan neuronal filament and DAPI was also evaluated. During the evaluation of peripheral nerve healing, inflammatory cell counts (absent: 3; between 1 and 9: 2; >10: 1), capillary counts (between 1 and 3: 3; between 3 and 6: 2; >6: 1), fibroblast and/or collagen status (normal structuring: 3; moderate: 2; intense: 1), endoneurial edema (minimal edema: 3; moderate: 2; extensive: 1), myelin sheath (circular and homogeneous: 3; vacuolar and weak: 2; absent or weak: 1), axonal continuity (full continuity: 3; moderate: 2; weak continuity: 1), and Schwann cell density (dense cell nuclei: 3; moderate density of nuclei: 2; few nuclei: 1) were examined [36–38]. Specimens were evaluated by histologists in a single blinded approach and higher scores represent favorable outcomes.

### 2.9. Thermal sensorial assessment

To evaluate the development of thermal sensorial threshold changes, the mice were placed in a clear Plexiglas chamber on an elevated mesh platform. The assessment involved applying a thermal stimulus with devices (YSED, Türkiye) heated to 50–56 °C or with a cold probe made by freshly delivered crushed dry ice loaded into a syringe and the latency for the mice to withdraw their hind paws was recorded to determine sensory responses [39].

### 2.10. Mechanical sensorial assessment

For mechanical sensorial testing, von Frey filaments (Ugo Basile, Italy) were used to apply graded mechanical stimuli to the plantar surface of the hind paws as previously described by Notartomaso et al. [40]. Briefly filaments were applied starting from one with a bending force of 0.008 g and were pressed perpendicularly to the plantar surface of the hind paw until it bent five times over a total period of 30 s. This procedure was conducted 5 times, with a 3-min interval between each application. A positive reaction was defined as a response to three out of five stimuli, with the corresponding filament being assigned as the pain threshold in grams.

### 2.11. Statistical analyses

GraphPad Prism Software 8 (GraphPad Prism Inc., USA) was used. One-way ANOVA was utilized for comparisons of single independent measurements of more than two groups and repetitive two-way ANOVA for repeated measurements. For nonparametric analysis, the Kruskal–Wallis test was chosen. Tukey, Bonferroni, and Dunn tests were used as post hoc tests for multiple comparison analyses. p < 0.05 was considered significant.

## 3. Results

### 3.1. HFCS consumption increased weight gain

**Figure 1:**
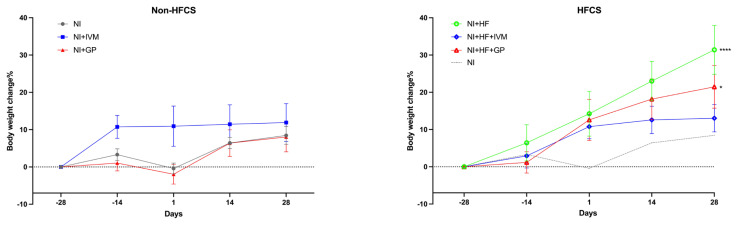
Mice’s body weight change over time. (a) There was no significant difference between groups that were not fed with HFCS. (b) Compared to the nerve injury group, the HFCS consuming groups’ body weight gain % was higher except for the NI+HF+IVM group. Data are shown as mean ± SEM. Statistical analysis was performed with two-way ANOVA and post hoc Bonferroni test (*p < 0.05; ****p < 0.0001 compared to the NI group) (n = 8/group).

We observed a significant difference in body weight change between the groups (F(5,200) = 6.83, p < 0.0001, Figure 1). The NI group gained 3.3 ± 1.6% with respect to the baseline until the SNI. Two weeks after the SNI, the animals exhibited a weight increase of 6.4 ± 1.5%; this increase reached 8.4 ± 2.4% by day 28. The NI+IVM group demonstrated weight gain of 10.8 ± 3% compared to its baseline, with no further increase observed between days −14 and 28 (Figure 1a). In contrast, the NI+HF group exhibited a linear weight gain (8.3 ± 1.2%) in every evaluation after day −14. In the HFCS groups (p values for NI+HF and NI+HF+GP groups were p < 0.001 and 0.005, respectively), body weight gain was higher with respect to that of the NI group except for the NI+HF+IVM group (Figure 1b).

### 3.2. IVM treatment resulted in faster recovery on rotarod tests in the HFCS groups

**Figure 2:**
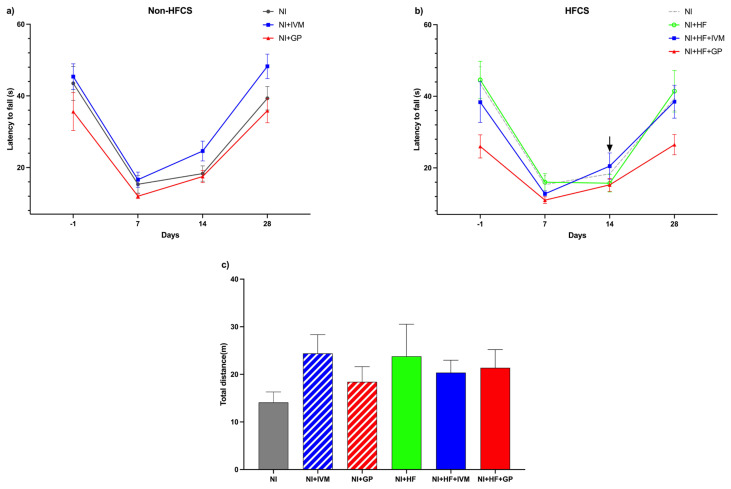
Variation in the latency to fall of mice on the rotarod test according to time and the total distances travelled in the OFA test. (a) There was no difference between the mean fall times of groups that were not fed with HFCS; when evaluated individually the NI, NI+IVM, and NI+GP groups showed shortened fall times after injury until day 28 (p < 0.05). (b) There was no difference between the mean fall times of the groups fed with HFCS; the NI+HF+IVM group showed no difference in fall time when compared to its own baseline on either day 14 or 28. The NI+HF and NI+HF+GP groups’ fall times were shorter compared to their baseline after injury until day 28 (p < 0.01). (c) There was no significant difference between total distance travelled on day 28. Data are shown as mean ± SEM. Statistical analysis was performed with two-way and one-way ANOVA and post hoc Bonferroni test; arrow indicates no difference compared to individual baseline (n = 6–8/group).

The latency to fall for all groups in the rotarod test was compared and a significant difference was found with respect to their baselines, which is day −1 (F (5,39) = 4.38, p < 0.005, Figures 2a and 2b). On day −1, NI group’s mean latency was 43.5 ± 4.7 s, 7 days after injury it dropped to its shortest time (15.3 ± 2.5 s, p < 0.05) among the selected time points, and on day 14 the mice showed improvement (18.3 ± 2.1 s, p < 0.05), but complete recovery was on day 28 (39.3 ± 3.3 s). The NI+HF+GP group’s baseline mean latency to fall was 26 ± 3.2 s, which is shorter than the NI group’s baseline. There were no significant differences in mean latency to fall among the groups. On day 7, all mice fell earlier than on day −1 (p < 0.05). By day 14 (20.5 ± 3.7 s), the NI+HF+IVM group data showed no difference compared to day −1 (38.4 ± 5.7 s). On day 28, the significant difference of all groups compared to their individual baselines disappeared.

### 3.3. OFA tests revealed no significant locomotory change at the end of the experiments

On day 28, the locomotion behaviors of the mice were measured to evaluate gross motor function by using the OFA test. The NI and NI+HF groups’ total distance travelled was 14 ± 2.2 m and 24 ± 6.7 m, respectively. There were no significant differences in the total distances traveled between the groups (Figure 2c).

### 3.4. IVM improved functional recovery on SSI measurements

**Figure 3:**
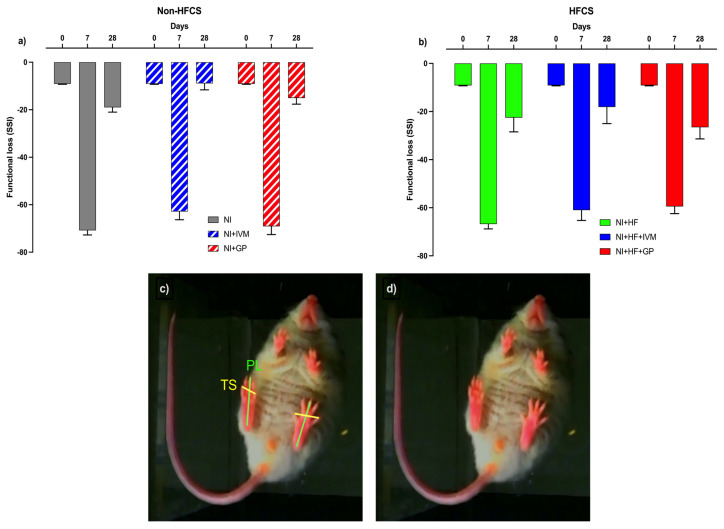
Static sciatic index results indicating loss of nerve function. (a) The NI+IVM group’s SSI on day 28 was higher compared to the NI and NI+GP groups. (b) There was no difference between the mean SSI of groups fed with HFCS. Data are shown as mean ± SEM. Statistical analysis was performed with two-way ANOVA and post hoc Tukey test (n = 5–8/group). (c) Toe spread (TS): distance between tips of the 1st and 5th toe, print length (PL): distance between tip of the 3rd toe and most posterior aspect of the paw in contact with the surface. (d) Demonstration of functional loss after SNI, seen as shortened distance between the 1st and 5th right toes of the mice.

Motor recovery was evaluated by SSI on days 0, 7, and 28 after SNI (Figures 3a–3d). Before the SNI, the index value of all groups was ~9.1 ± 0.2%. In the first week after the injury, SSI decreased to ~−65 ± 1.9% in all mice and increased to ~−18.4 ± 2.5% in week 4. The NI+IVM group’s 28-day SSI (−8.9 ± 2.7) was lower compared to that of the NI (−19 ± 2) and NI+GP groups (−15 ± 2.5) without statistical significance.

### 3.5. IVM treatment diminished HFCS-induced insulin increase and insulin resistance

**Figure 4:**
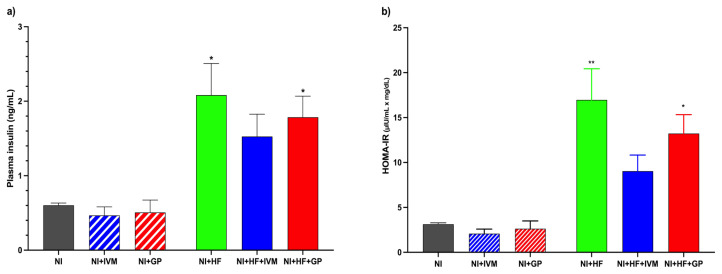
Plasma insulin levels and HOMA-IR values of the mice on day 28. (a) The NI+HF (p < 0.05) and NI+HF+GP (p < 0.05) groups had higher plasma insulin levels than the NI group. (b) Compared to the NI group, the NI+HF (p < 0.005) and NI+HF+GP (p < 0.01) groups had higher insulin resistance on day 28. Data are shown as mean ± SEM. For statistical analysis one-way ANOVA and post hoc Tukey test were used (*p < 0.05; **p < 0.01 compared to the NI group) (n = 4–7/group)

HFCS treatment yielded increased plasma insulin levels and insulin resistance except in the IVM group (F(5,26) = 7.4, p < 0.001). The increases in the NI+HF+IVM group’s insulin level and resistance were lower compared to those in the other HFCS groups. The NI group’s plasma insulin level was 0.6 ± 0.03 ng/mL. The NI+HF (2.1 ± 0.4 ng/mL, p < 0.05) and NI+HF+GP (1.8 ± 0.3 ng/mL, p < 0.05) groups’ plasma insulin levels were higher than those of the NI group (Figure 4a). HOMA-IR comparisons revealed a significant difference between the groups (F(5,26) = 12.93, p < 0.0001). Similar to insulin levels alterations, the NI+HF (17 ± 3.5) and NI+HF+GP (13 ± 2.1) groups displayed higher insulin resistance compared to the NI group (3.1 ± 0.16, p < 0.005, 0.01, Figure 4b).

### 3.6. IVM showed better healing scores in histopathological evaluations

**Figure 5:**
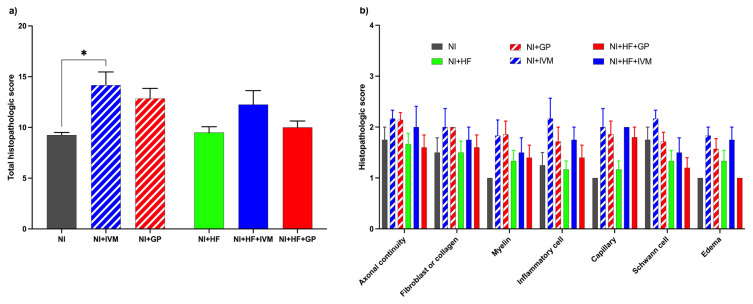
Total histopathological and subhistopathological score analysis results of injured sciatic nerves of mice on day 28. (a) The NI+IVM group had a significantly higher total histopathological score compared to the NI group at the end of follow-up (p < 0.05). For analysis the Kruskal–Wallis test and post hoc Dunn’s test were used (*p < 0.05 compared to the NI group) (n = 4–7/group). Data are shown as mean ± SEM. (b) The distribution of subscores for all groups.

Total histology scores differed between the groups (Kruskal–Wallis statistic = 13.78, p = 0.017). The total score of the NI+IVM group was higher than that of the NI group (mean rank difference −16.04, p < 0.05, Figure 5a). The histopathological subscores are presented in Figure 5b.

### 3.7. IVM exhibited accelerated healing but not in HFCS fed mice

**Figure 6:**
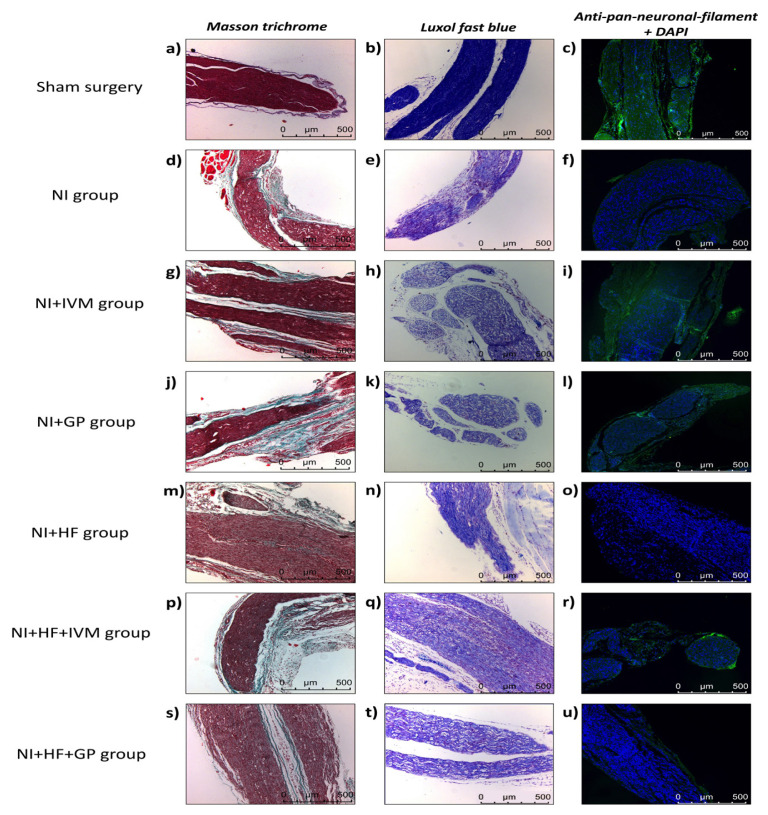
Histology of mice sciatic nerves. (a, d, g, j, m, p, s) Masson’s trichrome staining demonstrates connective tissue elements (i.e. collagen, fibroblasts), blood cells, and capillaries. (b, e, h, k, n, q, t) Luxol fast blue staining exhibits myelin intensity and distribution in blue and tissue edema as vacuolar structures. (c, f, i, l, o, r, u) Anti-pan-neuronal filament+DAPI staining expresses Schwann cell nuclei as blue, neurofilaments as green.

The sham surgery group (Figures 6a–6c) showed organized nerve fibers and a dense and continuous myelin sheath with no signs of inflammation or edema. In the NI group (Figures 6d–6f), Masson’s trichrome staining revealed a dense accumulation of fibroblasts and collagen fibers within the nerve connective tissue. Additionally, inflammatory cells and capillaries were prominently observed. Notable edema surrounded the nerve fibers, and the myelin sheath displayed nonhomogeneous continuity, manifesting as vacuolar structures. In Luxol fast blue stained preparations, we observed reduced or absent staining, suggesting areas of myelin degeneration or loss. The NI+IVM and NI+GP groups exhibited improved histological organization with respect to the NI group. The NI+IVM group showed less edema than the NI+GP group, indicating a faster healing process.

In the NI+IVM group (Figures 6g–6i), the reduced number of inflammatory cells and capillaries, along with well-organized fibroblast and collagen structures, indicated a more advanced stage of healing. Moderate edema and minimal vacuolization were still observed among the nerve fibers. Fluorescent labeling revealed moderately increased intensity of Schwann cell nuclei, visualized by DAPI and increased neurofilament synthesis.

In the NI+GP group (Figures 6j–6l), Masson’s trichrome staining revealed moderate fibroblast and collagen organization, but a higher density of inflammatory cells and capillaries than in the IVM group. Additionally, pronounced edema indicated an earlier stage of healing in this group. Myelin continuity was also affected due to the intense edema.

In the NI+HF group (Figures 6m–6o), excessive numbers of inflammatory cells and capillaries were observed. Due to severe edema, the nerve fibers were separated, leading to a disruption. Additionally, nerve tissues exhibited a widespread presence of lipid-storing cells in the NI+HF group (Figure S1).

### 3.8. IVM treatment improved thermal (hot) sensorial recovery in the HFCS groups

**Figure 7:**
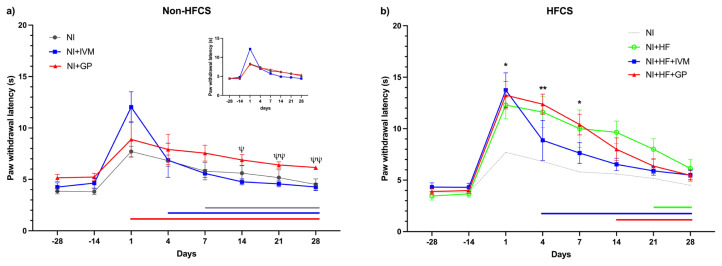
The mice’s hind paw withdrawal latencies representing hot sensorial function. (a) The NI+IVM group had shorter mean paw withdrawal latencies compared to the NI+GP group 14 days after injury until the end of follow-up, which disappeared after data normalization (inset graph). The NI+GP group exhibited no difference compared to its baseline through follow-up. (b) Compared to the NI group, the NI+HF+GP group had longer paw withdrawal latencies after injury until day 7. When compared to its own baseline value the NI+HF+IVM group showed no difference on day 4 until the end of follow-up. The NI+HF group had longer withdrawal response compared to its own baseline on days 1, 4, and 14; the NI+HF+GP group had longer withdrawal latencies on days 1, 4, and 7. Data are shown as mean ± SEM. For analysis two-way ANOVA and post hoc Tukey and Bonferroni tests were used. Horizontal lines with respective colors represent no significant difference compared to its individual baseline (*, ψ p < 0.05; **, ψψ p < 0.01 compared to the NI and NI+IVM groups, respectively) (n = 7–8/group).

In the hot sensorial tests, we observed significant increases in paw withdrawal latencies among the groups following SNI (F(5,38) = 2.8, p < 0.05). The NI group displayed significantly increased latencies for 7 days (p < 0.01) after the injury and it returned to the baseline afterwards. Statistical insignificance in latencies occurred on day 4 for the IVM group and the gabapentin group did not show significant alteration following SNI (Figure 7a). Since the baseline for the NI+GP group was significantly higher than that of the others, we also evaluated the data by normalizing according to their individual baselines and ran the comparisons again. Before normalization, the NI+GP group exhibited increased paw withdrawal latencies compared to the NI+IVM group across days 14, 21, and 28 following the injury (p < 0.05, <0.01, <0.05), which disappeared after normalization (Figure 7a). The NI+HF+GP group exhibited longer latencies compared to the NI group during the first week following SNI [day 1 (8.3 ± 0.5 s vs. 13.2 ± 1.3 s), p < 0.05; day 4 (7.4 ± 0.5 s vs. 12.4 ± 1 s), p < 0.01; day 7 (6.4 ± 0.8 s vs. 10.4 ± 1 s), p < 0.05]. At the end of the experiments, responses to hot stimuli returned to their baselines in all HFCS groups. Drug treatment shortened this period to 4 and 14 days for IVM and gabapentin, respectively (Figure 7b). The HFCS fed groups’ withdrawal latencies were higher than the NI group’s without statistical significance.

### 3.9. IVM treatment improved thermal (cold) sensorial recovery in the non-HFCS groups

**Figure 8:**
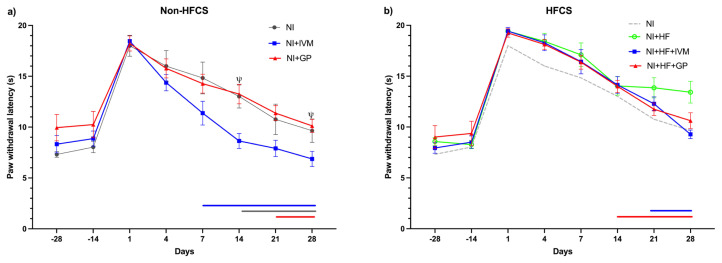
The mice’s hind paw withdrawal latencies representing cold sensorial function. (a) The NI (p < 0.05) and NI+GP (p < 0.01) groups had longer paw withdrawal latencies after injury until days 7 and 14, respectively. The NI+IVM group returned to its baseline by day 4 (p < 0.05). The NI+IVM group had shorter mean paw withdrawal times on days 14 and 28 compared to the NI+GP group. (b) The NI+HF (p < 0.05) and NI+HF+IVM (p < 0.01) groups had longer withdrawal latencies after injury until days 28 and 21, respectively. The NI+HF+GP group returned to its baseline by day 7 (p < 0.01). Data are shown as mean ± SEM. Statistical analysis was performed with two-way ANOVA and post hoc Tukey and Bonferroni tests; horizontal lines with respective colors represent no significant difference compared to its individual baseline (ψ p < 0.05 compared to the NI+IVM group) (n = 7–8/group).

In the cold sensorial tests, all mice exhibited decreased responses following SNI (F(5,38) = 4.445, p < 0.005), which became evident by the increased paw withdrawal latencies. In all the non-HFCS groups of mice, decreased responses to cold stimuli completely recovered in 28 days (Figure 8a). The NI, NI+IVM, and NI+GP groups displayed significantly decreased response to cold until days 14, 7, and 21, respectively (p < 0.05). Mean withdrawal latencies were slightly higher in the HFCS mice than in the non-HFCS groups after SNI (Figure 8b). In NI+HF group, latencies did not return to the baseline after day 28, showing incomplete recovery. All animals treated with IVM and gabapentin returned to baseline values at 28 and 14 days. The cold sensorial data indicated that the NI+IVM group had reduced paw withdrawal latencies on days 14 and 28 after injury compared to the NI+GP group (p < 0.05).

### 3.10. IVM improved mechanical thresholds faster than all other groups following nerve injury

**Figure 9:**
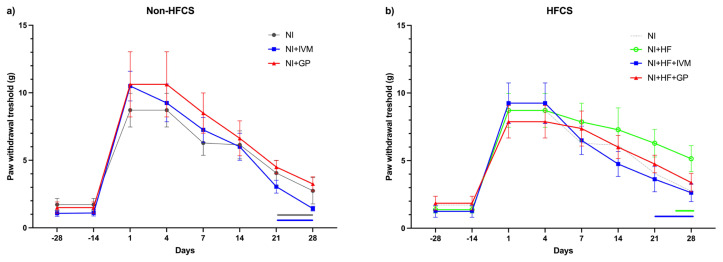
Hind paw mechanical thresholds of the mice tested with von Frey filaments. (a) Both the NI and NI+IVM groups had higher paw withdrawal thresholds compared to their baselines (14 days before injury) after injury until day 14 (p < 0.05; <0.05). The NI+GP group had higher paw withdrawal thresholds compared to its baseline after injury until the end of follow-up on day 28 (p < 0.05). (b) Compared to its baseline the NI+HF+IVM group had higher mechanical thresholds after injury until day 14 (p < 0.05). The NI+HF group had higher thresholds until day 21 (p < 0.05). The NI+HF+GP group showed higher thresholds after injury compared to its baseline until the end of follow-up on day 28 (p < 0.05). Data are shown as mean ± SEM. Statistical analysis was performed with two-way ANOVA and post hoc Bonferroni test. Horizontal lines with respective colors represent no significant difference compared to its individual baseline (n = 7–8/group).

Average mechanical thresholds were similar between the groups. All groups exhibited significant changes in their paw withdrawal thresholds compared to their respective baselines. Thresholds comparisons of the NI and NI+IVM groups with their individual baselines following SNI showed significant elevation for 21 days (p < 0.05). In the gabapentin-treated group elevation was significant until day 28 (p < 0.001, Figure 9a). In the NI+HF group, paw withdrawal thresholds were slightly higher than those in the NI group and did not return to the baseline, indicating incomplete recovery. In the NI+HF group, the threshold returned to the baseline on day 28, showing incomplete recovery. All animals treated with IVM returned to baseline values on day 21. Gabapentin treated animals’ thresholds did not recover to baseline values even on day 28 (Figure 9b).

## 4. Discussion

In the present study, we investigated the effects of IVM and HFCS consumption on nerve repair/regeneration in a mouse model of SNI. Chronic IVM treatment significantly shortened the time to regain neurological functions and improved histological parameters. Eight weeks of HFCS consumption did not influence motor recovery but partially impaired sensorial healing and caused poor nerve healing histologically.

By day 28, the locomotion, gross motor function, and gait of all animals had returned to their baselines. Although no differences in final motor recovery were observed between the IVM-treated and untreated groups after 28 days, we cannot discount the possibility of changes in fine motor activity. The SSIs of the IVM-treated mice were lower, indicating better sciatic nerve function at day 28 compared to other groups but the difference was not statistically significant.

In the present study, we also found that IVM did not change locomotion after 4 weeks of treatment, similar to previous research [41]. Since we did not observe any significant changes in the motor function of animals following chronic treatment with IVM, a GABAergic inhibitory drug, we concluded that the administered dose did not cross the blood–brain barrier and did not lead to adverse effects in the central nervous system. Therefore, our findings support the idea that IVM’s effects are more pronounced on peripheral nerves rather than the central nervous system [42]. Based on our rotarod results, the HFCS group treated with IVM showed faster recovery in terms of gait, with improvements occurring nearly 2 weeks earlier. Gabapentin did not alter the course of healing in either the HFCS or non-HFCS groups. It was shown that IVM exhibits antiinflammatory actions and improves neuronal healing by promoting regenerative processes via regulating NF-κB, Treg/Th17 cell balance, IFN-γ, and IL-17A [43–45]. The performance on the rotarod is a valuable indicator of neurological deficits, as it demands a combination of proprioceptive, vestibular, and precise motor skills for balance. The faster recovery associated with IVM could be attributed to these features.

High fructose treatment yielded significantly higher plasma insulin levels and HOMA-IR scores but not in the IVM-treated HFCS group. Although high fructose was able to increase insulin levels and the HOMA-IR score to some extent in the NI+HF+IVM group, it was still less than in the other HFCS groups, including the gabapentin group, indicating a role of IVM in glucose metabolism and/or insulin sensitivity. Consistent with the finding of our study, Jin et al. suggest that IVM reduces insulin resistance by regulating insulin sensitivity via the Farnesoid X receptor [46]. Similarly, all HFCS consuming animals exhibited higher body weight gain [47] compared to the NI group except the IVM treatment group. One of the well-known outcomes of insulin resistance is obesity. It is reasonable to consider that a treatment thought to influence insulin metabolism might likewise inhibit weight gain [48].

On day 28, all groups exhibited similar motor improvements, but histological healing was not completed. Histopathologic evaluation of the sciatic nerves showed that IVM led to higher total histologic scores, demonstrating better healing. This may be due to IVM’s effect on fibroblasts to induce the transformation into glia-like cells and increase the release of glial cell-derived neurotrophic factor (GDNF) during wound healing [27], indicating that IVM may promote the repair and regeneration of peripheral nerves. Additionally, gabapentin slightly improved histological recovery. We also observed a similar trend for IVM in the histological scores of the HFCS groups. With the burden of HFCS consumption, the antiinflammatory and neurotrophic effects of IVM seem to be minimized.

Since uncontrolled inflammation stunts the healing process, the antiinflammatory properties of IVM may also facilitate nerve regeneration. Xie et al. showed antiinflammatory properties of IVM in an experimental autoimmune encephalomyelitis (EAE) model. The mechanism/s may involve maintenance of Treg/Th17 cell balance and inhibition of secretion of cytokines such as IFN-γ and IL-17A [43]. Moreover, IVM is an allosteric modulator of P2X4 receptor present in various immune cell types and endothelia [44]. With IVM binding P2X4 receptor signaling may promote an antiinflammatory state in microglia and macrophages, may enhance the phagocytic removal of damaged myelin, facilitating the remyelination process and improving the clinical symptoms of EAE [45].

We consistently observed loss of sensory nerve function after injury in thermal and mechanical sensation. IVM treatment significantly accelerated the recovery of heat sensation, while gabapentin showed no notable impact on the timeline of recovery in the HFCS groups. IVM treatment resulted in a faster recovery of cold sensation in the non-HFCS groups as well.

In the assessment of mechanical sensation, gabapentin treatment failed to restore baseline thresholds in either the non-HFCS or HFCS groups. On the other hand, in the HFCS groups, IVM treatment demonstrated a faster recovery. The diverse responses observed across tested sensory modalities may be attributed to receptor level differences (i.e. Merkel disks, cold-specific, warm-specific receptors) or primary afferent nerve differences including axonal diameter, conduction velocity, etc. The discrepancy in our thermal latency results may be a consequence of different numbers of nerve endings, as cold sensitive endings are ~5 times greater than warmth sensitive endings [49].

In the present study, the HFCS groups displayed decreased response to thermal stimuli compared to the NI group. Furthermore, they were significantly slower in regaining their thermal and mechanical sensorial functions compared to the non-HFCS groups. This shows the negative outcomes of high fructose even in an 8-week period with a relatively low dose. Hyperglycemic insulin resistant metabolic state leads to inhibition of fibroblast proliferation and resistance to growth factors [50]. As shown for fibroblasts, high sugar levels and glycated by-products have a toxic effect on endothelial cells and the vascular walls [51]. Subsequently, these pathological processes may lead to disruption of the peripheral nerve healing process.

One limitation of our study is that it did not explore other doses of IVM treatment, which could lead to better outcomes. Additionally, using only male mice might miss important sex-based differences in response to HFCS.

The results suggest that IVM, a drug that is already in current medical use, may have potential therapeutic benefits via regeneration promoting properties. Additional research is required to evaluate IVM’s potential to accelerate healing following physical nerve injuries. Exposure to HFCS over a 2-month period resulted in slower and incomplete healing in sensorial and motor nerve functions. Additionally, IVM administration also appeared to mitigate HFCS-induced insulin resistance, suggesting a potential protective effect against metabolic disruptions associated with high fructose intake.

## Supplementary Figure

Figure S1 Regardless of nerve injury, lipid-storing cells were widely observed in the nerve tissue of HFCS-fed mice.
In the NI+HF group, excessive number of inflammatory cells and capillaries were observed. Due to severe edema, the nerve fibers were separated, leading to a disruption. Additionally, nerve tissues exhibited a widespread presence of lipid-storing cells in the NI+HF group.

## Figures and Tables

**Table:**
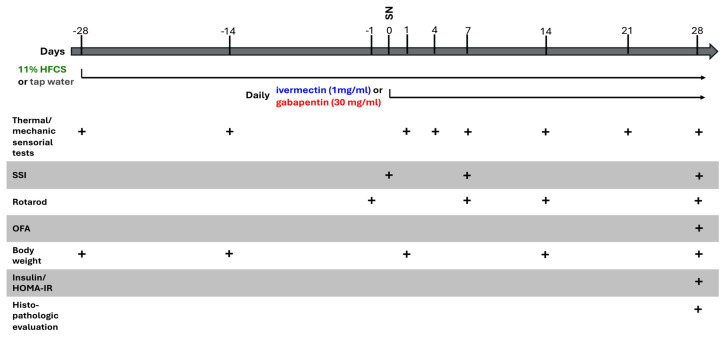
Study flowchart, SNI: sciatic nerve injury, SSI: static sciatic index, OFA: open field arena test, HFCS: high fructose corn syrup, HOMA-IR: homeostatic model assessment for insulin resistance.
