# Supplementary figures and images for: Impact of ivermectin on nerve regeneration following sciatic injury in mice: the consequences of dietary high fructose
Source: Turk J Med Sci. 2024 Dec 19;55(1):299–312. doi: 10.55730/1300-0144.5971 (PMC11913497; doi:10.55730/1300-0144.5971)

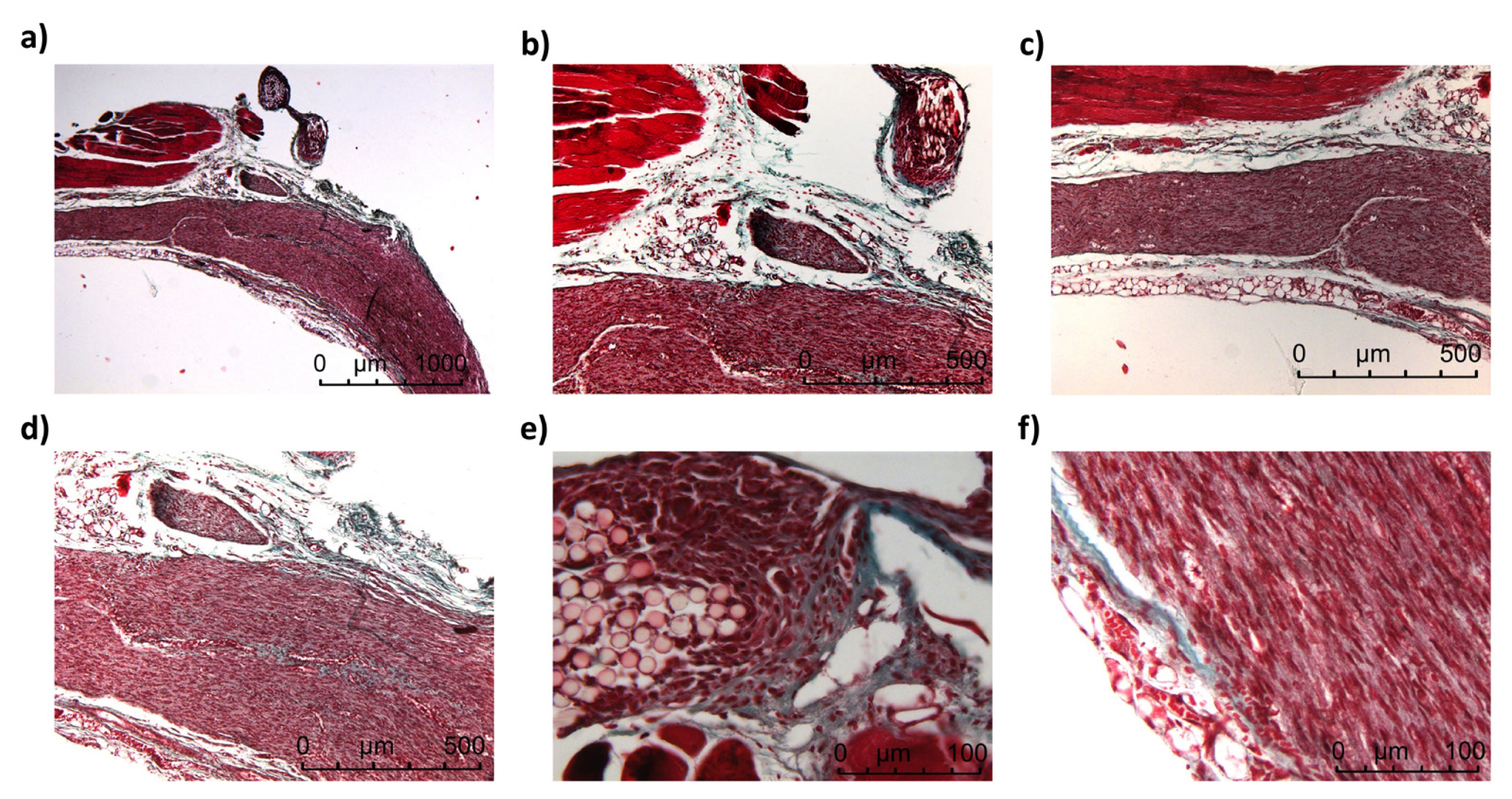

Supplement: Figure S1 — Regardless of nerve injury, lipid-storing cells were widely observed in the nerve tissue of HFCS-fed mice. In the NI+HF group, excessive number of inflammatory cells and capillaries were observed. Due to severe edema, the nerve fibers were separated, leading to a disruption. Additionally, nerve tissues exhibited a widespread presence of lipid-storing cells in the NI+HF group. [file tjmed-55-01-299s1.tif]
